# Negative pressure wound therapy combined with skin grafting improves surgical wound healing in the perianal area

**DOI:** 10.1097/MD.0000000000004670

**Published:** 2016-09-02

**Authors:** Shi Jia-zi, Zhai Xiao, Li Jun-hui, Xue Chun-yu, Bi Hong-da

**Affiliations:** a Graduate Management Unit, Second Military Medical University; b Graduate Management Unit, Second Military Medical University; c Department of Plastic Surgery, Changhai Hospital, Second Military Medical University, Shanghai, China.

**Keywords:** negative pressure wound therapy, perianal tumor, perianal wound, skin grafting

## Abstract

Management of large tissue defects resulting from local wide resection of perianal is a clinical challenge for surgeons. The aim of the present study was to investigate the efficacy of negative pressure wound therapy (NPWT) following skin grafting on perianal surgical wound healing.

Included in this study were 12 patients with perianal tumors who received skin grafting after perianal tumor resection between December 2012 and December 2014. A self-designed negative pressure drainage device was then applied to maintain a standard negative pressure at −150 mm Hg and removed on day 8 postoperation. The outcome was recorded immediately after NPWT and at 6-month follow-up.

All skin grafts survived without infection, hematoma, and necrosis in all 12 patients. No tumor recurrence was detected during 6-month follow-up. Natural folds were observed around the anus. All patients showed normal bowel movements.

NPWT following skin grafting was effective for perianal surgical wound healing and infection prevention, thus benefiting anatomical and functional recovery of the anus.

## 1 Introduction

Some locally advanced tumors such as Bowen disease, perianal Paget disease, and basal cell carcinoma often invade crissum epidermis.^[1]^ A wide resection of skin required in such cases often results in large tissue defects,^[2]^ and repair of these perianal defects remains a big clinical challenge.^[3]^ It is now widely believed that skin grafting is important for covering the defects and restoring anal function during the construction.^[4]^

However, skin grafting is contraindicated without construction of a temporary anus, or it may run a high risk of infection and necrosis because of long-term confinement, anal stenosis caused by graft contracture, and ectropion. A covering colostomy may be performed in about 20% patients, which often causes abdominal scarring, increased pain, intestinal adhesion, and other complications.^[5]^ Furthermore, the skin near the anus is so thin and flexible that a thick flap maybe less suitable than a thin skin graft for simulating the nature structure.^[6]^ However, a perianal skin graft is prone to fail due to the irregular wound surface there. Negative pressure wound therapy (NPWT) is an established and effective tool for surgical wound management, knowing that it facilitates wound healing through increasing blood flow, reducing edema and infection, and promoting tissue granulation.^[7]^ To the best of our knowledge, few studies have reported combination of NPWT and skin grafting for perianal wound management.

In the present study, we designed a simple negative pressure drainage device and used it to close the anus and press against the grafts firmly in 12 patients with perianal tumors who received NPWT and skin grafting after extensive tumor resection, with satisfactory therapeutic outcomes.

## 2 Methods

**Figure 1:**
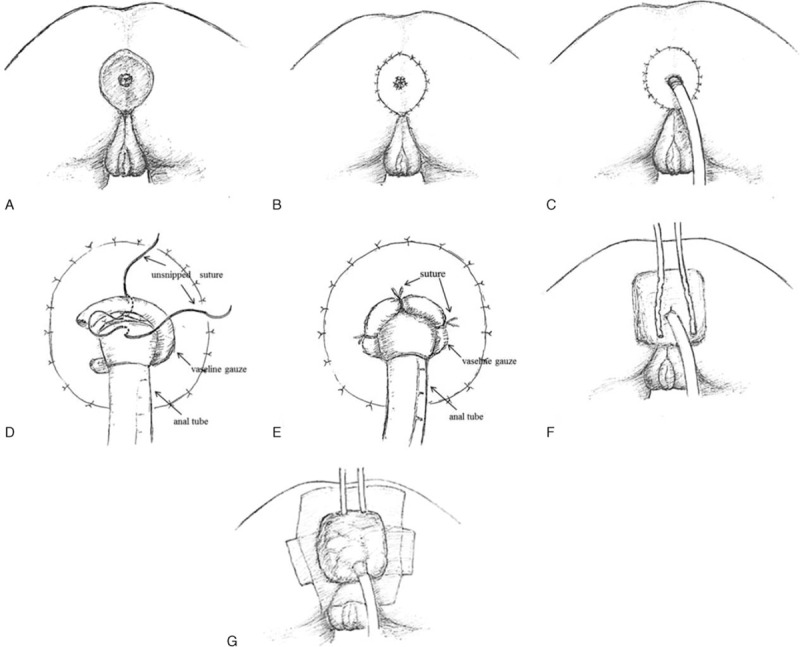
Negative pressure wound therapy pattern diagrams. (A) A circular wound was formed after removing the lesion; (B) repairment of the wound with an intermediate split thickness skin graft; (C) insertion of the anal tube and packing of the anus with vaseline gauzes; (D) 4 to 5 sutures were left unsnipped as suturing the skin graft with the perianal skin outside; (E) the preserved sutures were sutured with the vaseline gauzes twining around the anal tube; (F) aseptic dressings were placed on the surface of the graft, and 2 drainage tubes were indwelt in the dressings; and (G) after closing the dressings, the drainage tubes and skin with a sticky film were connected to −150-mm Hg negative pressure source.

Included in this study were patients with perianal wounds after tumor resection performed between December 2012 and December 2014. Demographic data of the patients were collected. Before operation, carcinoembryonic antigen was detected, and abdominal Computed Tomography and colonoscopy were performed. Patients with metastasis and associated tumors were excluded. All patients provided written informed consent about skin grafting and postoperative NPWT. All surgical procedures were performed by the same surgeon in a single team as follows:

1. Tumor resection: the patient was placed in a lithotomy or jackknife position. An extensive resection (0.5–1.5 cm expansion from the lesion margin was defined before the surgery. However, the final surgical margin was determined according to intraoperative frozen pathology result) was conducted after the combined spinal epidural anesthesia. After removing the lesion along the sphincter for anal function preservation, local biopsy was performed to confirm the intradermal lesion. If the tumor invaded tissues above the dentate line, a mucosa dissection was necessary (Fig. 1A).
2. Skin grafting: depending on the wound size, an appropriate intermediate split thickness skin was cut from the patients’ buttock or abdomen, with the central tissue of the graft cut off. Then, a circular hole was formed. The graft was used to cover the defect and secured by suturing it with the rectal mucosa inside and the perianal skin outside (Fig. 1B).
3. Placement of the anal tube and the negative pressure drainage device:
  1. Choose a suitable anal tube and insert it into the anal canal at a depth about 12 cm (Fig. 1C).
  2. Use vaseline gauzes to twine the anal tube at the joint part with the anus. The skin graft was sutured with the perianal skin outside with 4 to 5 sutures left unsnipped (Fig. 1D). Then the preserved sutures were sutured with the vaseline gauzes twining around the anal tube for the sake of minimizing the slit between anal tube and mucosa (Fig. 1E). The other spaces were filled out subsequently, and the grafted skin was protected with absorbent cotton to ensure a tight closure.
  3. Drainage tubes were placed in the dressings and connected to a commercially available machine (Kinetic Concepts Inc. [KCI], San Antonio, TX) to generate vacuum source. The machine from KCI can automatically monitor pressure change to maintain a constant pressure. The vacuum was most commonly set to −150 mm Hg. Three to 5 layer dressings were paved above and sealed with sticky films (Fig. 1F and G).

All patients took an active position and were advised to eat liquid diet and do moderate exercise postoperatively. The dressings were opened for examination on day 4 postoperation, and on day 8 the device was removed to check for survival of the graft.

Data regarding the diagnosis, the length of hospital stay, complications, and the survival rate of grafts were recorded after therapy. Tumor recurrence or metastasis, long-term complications, and the condition of grafts were also observed during a 6-month follow-up period.

For statistical analysis, data were presented as mean ± standard deviation. Since it was a case series, no further comparison was analyzed.

## 3 Results

**Figure 2:**
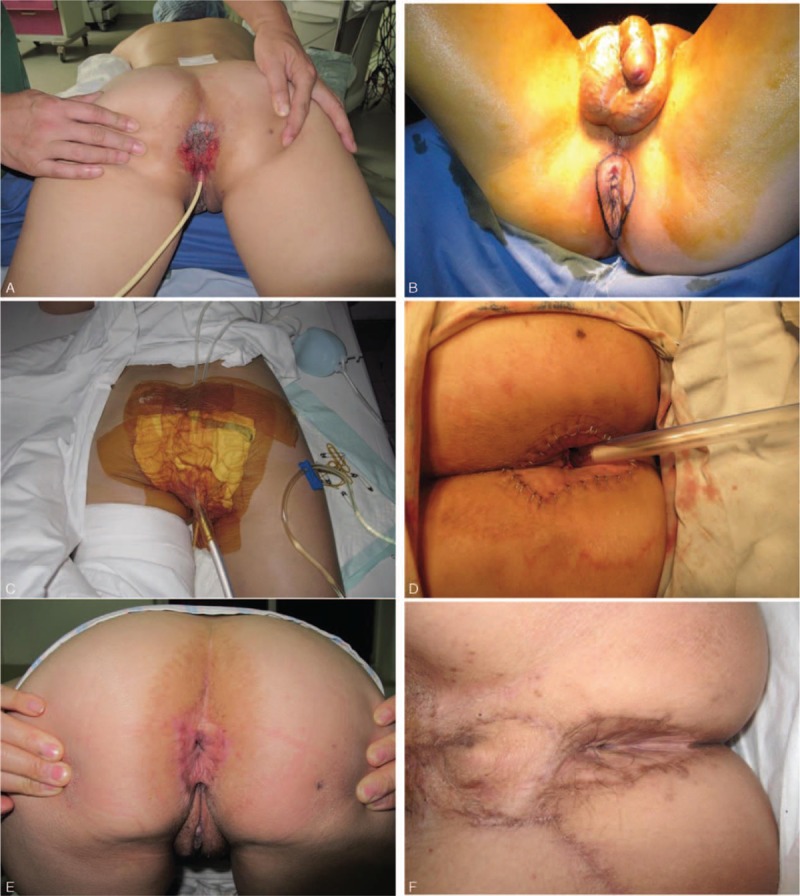
(A and B) Perianal Paget disease before treatment, (C and D) negative pressure wound therapy treatment after skin grafting, (E and F) 6 months after therapy. Two patients were included: a 57-year-old female (A, D, and E) and a 63-year-old male (B, C, and F).

Twelve patients (7 males and 5 females) were totally included in the present case series. Demographics, diagnosis, and hospitalization are summarized in Table 1. The mean age was 61.5 ± 4.84 years. Three patients had a history of hypertension. No other clinical history was elicited. All 12 patients received surgical therapy. The histological diagnosis was Paget disease in 8 cases, Bowen disease in 3 cases, and basal cell carcinoma in 1 case. The mean maximum diameter of these surgical wounds was 7.0 ± 1.5 cm. Dressing pollution was observed in 1 case on day 4 postoperation. Routine scavenging, dressing change, and compression were performed. No significant complication occurred. All skin grafts survived on day 8 postoperation. No local scar was observed during the 6-month follow-up period. The grafted skins were soft and able to shrink to close the anus. No incontinence, tumor recurrence, or metastases occurred (Fig. 2A–F).

**Table 1:**
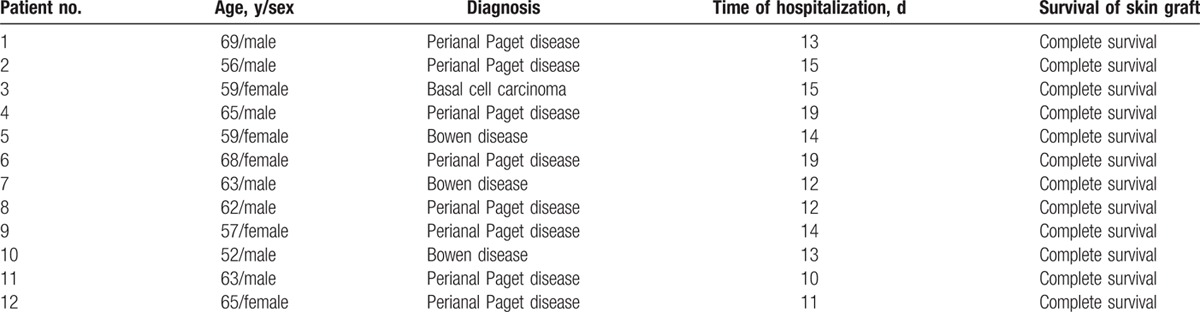
Patients’ information and survival of the skin grafts.

## 4 Discussion

Tumor resection for perianal lesions often results in injury to the dental line, which is a complex structure between the rectal mucosa and dermis, and plays an important physical role. Extensive excision involving the dental line often leaves a large defect for patching, which is a cosmetic and functional challenge for surgeons.^[8]^ This is the first report describing combination of the artificial anal tube with NPWT after skin grafting to improve surgical wound healing in the perianal area.

The anal sphincter fibers that terminate in the subcutaneous tissue play the role of contracting the perianal skin shrinks and closing the anus. The reestablishment of the connection between sphincter and dermis is helpful for the functional recovery.^[9]^ Nevertheless, skin grafts often fail to survive because of irregularity of the wound, infection, and other factors. Colostomy is a routine therapy used to redirect stool and reduce local pollution. In such a case, at least 2 to 3 operations are needed. Complications such as intestinal adhesion and abdominal scarring occur occasionally, which greatly increase the postoperative pain. In addition, secretion of the intestinal gland cannot be avoided and often results in local pollution.^[10]^ Compared with skin grafts, skin flaps with subcutaneous fat tissue are relatively thick, which may be against dynamic reconstruction between the sphincter and dermis.^[11]^ At any rate, there exists a difference in circumference and elasticity between the grafted skin and the perianal wound. Postoperative scar formation also goes against anal contraction.

NPWT has been widely used. Some studies indicate that the presence of negative pressure can effectively increase blood flow, clear exudates, and facilitate wound healing.^[12]^ In our self-designed device, we used anal tube for stool drainage. Negative pressure might guarantee the local closure and prevent intestinal infection. The grafted skin was definitely compressed. Primary healing was achieved in all grafts of our study. During the 6-month follow-up period, we found that the skin grafts were well elastic and able to shrink casually with the drive of the sphincter.

The technique applied in this study has several advantages as follows: first, it avoids using laparotomy which may cause abdominal scar formation and intestinal adhesion. Second, patients could eat liquid diet after surgery instead of totally parenteral nutrition, thus reducing the nutritional support cost. Third, all procedures were completed in 1-stage operation, thus shortening the treatment course. Fourth, the operative technique and postoperative treatment are relatively simple and easy. Lastly, all the materials in our device are clinically accessible and available at relatively low costs.

Although the present study is a single case series with no control group, we still believe that this method used in rare disease entity might get satisfied outcomes. It would be an alternative therapy to be popularized.

## 5 Conclusion

NPWT directly applied with skin grafting for perianal wounds is effective in preventing infection, improving wound healing. It was beneficial for anatomical and functional recovery of the anus.
